# Contribution of magnetic resonance imaging in the diagnosis of talus skip metastases of Ewing's sarcoma of the calcaneus in a child: a case report

**DOI:** 10.1186/1752-1947-5-451

**Published:** 2011-09-12

**Authors:** Hicham Jalal, Zoubida Belhadj, Hind Enneddam, Mohammed Madhar, Tarik Fikry, Omar Essadki, Ahmed Ousehal

**Affiliations:** 1 Department of Radiology, Ibn Tofail Hospital, Gueliz, Marrakesh, 40000, Morocco; 2 Department of Traumatology, Ibn Tofail Hospital, Gueliz, Marrakesh, 40000, Morocco

## Abstract

**Introduction:**

Ewing's sarcoma of the calcaneus is rare. About thirty cases with calcaneus involvement have been reported in the literature. Talus skip metastases have rarely been described in the available literature

**Case presentation:**

We report a case of a 14-year-old Moroccan boy, who presented with Ewing's sarcoma of his right calcaneus, diagnosed by swelling of the calcaneus evolving over a year. Radiography, computed tomography and magnetic resonance imaging showed an important tumoral process of the calcaneus and talus skip metastases. The diagnosis was confirmed with histology after a biopsy. In spite of amputation and postoperative chemotherapy, our patient died six months later due to secondary respiratory distress after lung metastasis.

**Conclusion:**

Imaging, especially magnetic resonance, is important in the diagnosis of Ewing sarcoma and skeletal skip metastases. Treatment of Ewing's sarcoma consists of chemotherapy, radiation therapy and surgical resection depending on the stage and extent of the disease. With the exception of lesions in the calcaneus, the prognosis for disease-free survival of Ewing's sarcoma of the foot is excellent.

## Introduction

Ewing's sarcoma is a rare malignant bone tumor that may affect any bone, usually occurring in long bones, pelvis and ribs, with only 3-5% of cases in the bones of the hands and feet [1]. It is a highly anaplastic round-cell tumor, primarily arising in the intramedullary portion of the bone.

### Case presentation

A 14-year-old Moroccan boy presented with painful swelling of his right foot of 12 months duration. A general examination was unremarkable, while local examination revealed a diffuse swelling involving his right ankle joint and foot. The overlying skin was normal. The swelling was tender and mobility at the joint was restricted. Hematological and biochemical investigations revealed a normal hemogram and normal liver and renal function tests.

**Figure 1:**
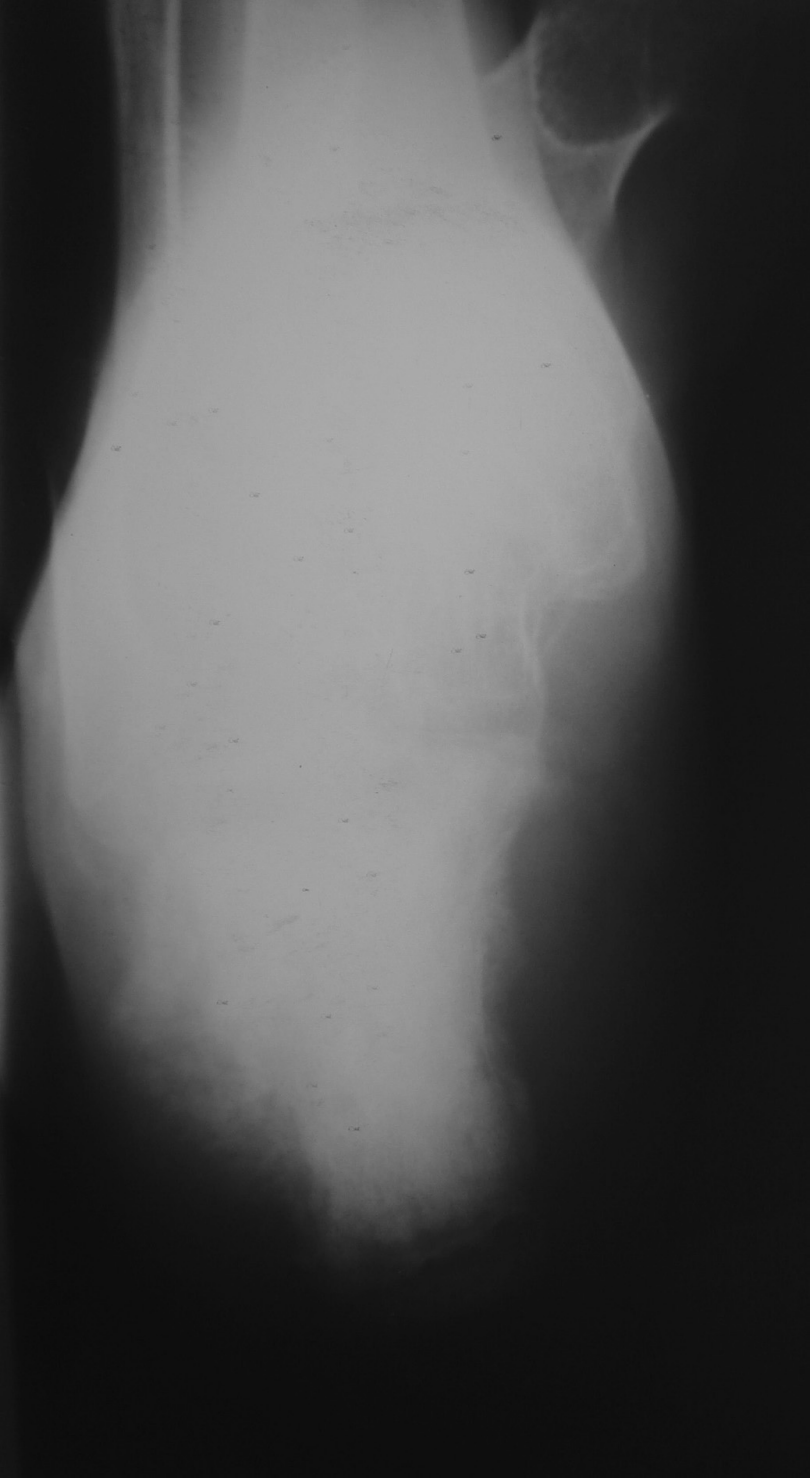
Anteroposterior radiographs of patient's foot show a lesion in the calcaneus condensed with aggressive periosteal reaction and soft-tissue swelling.

**Figure 2:**
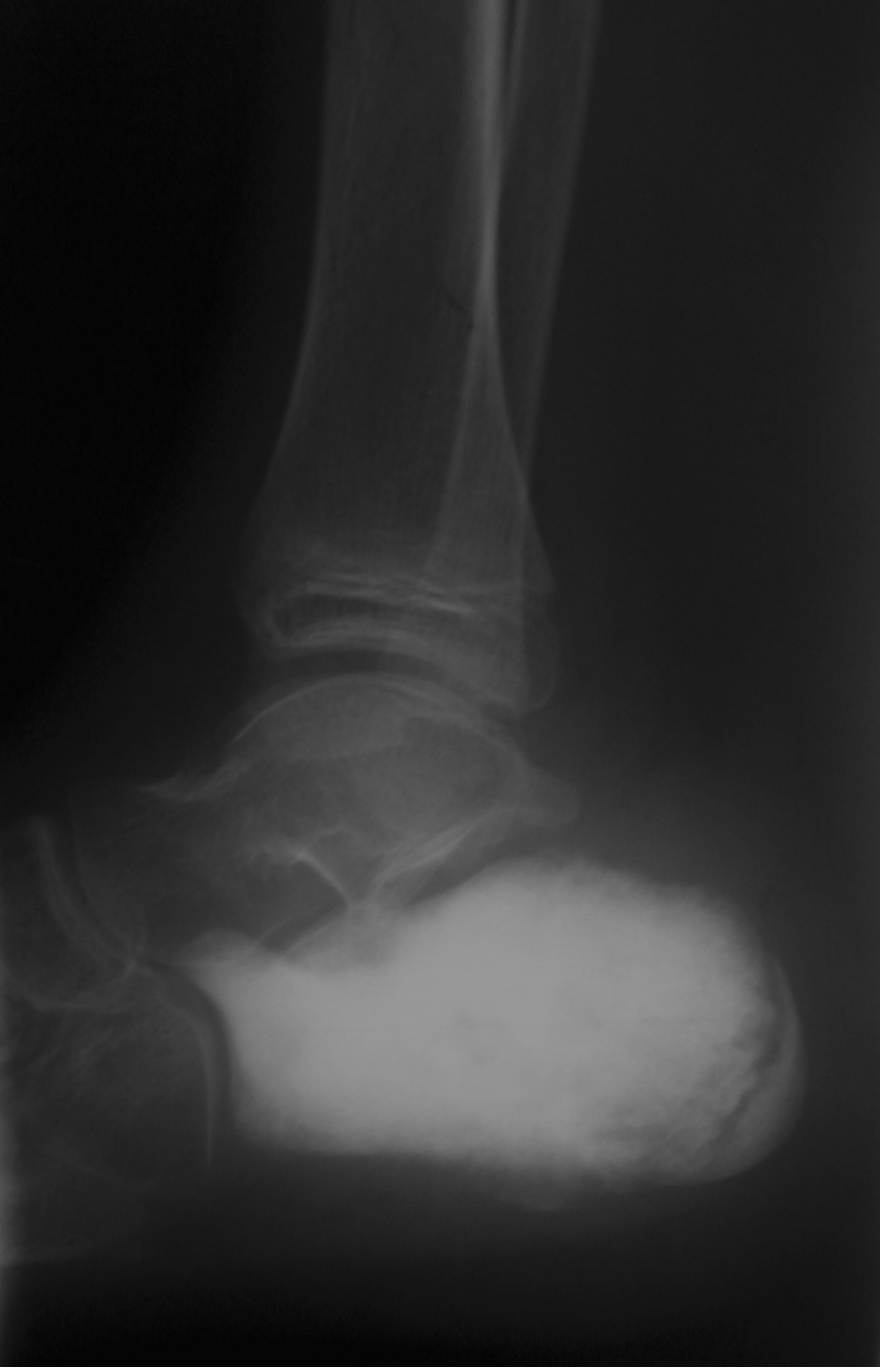
**Lateral radiographs of the patient's foot show a condensed lesion in the calcaneus with aggressive periosteal reaction and soft-tissue swelling**.

**Figure 3:**
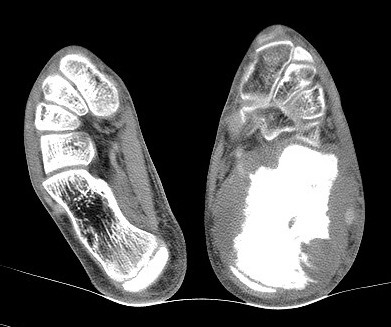
**CT image of the patient's foot, revealing a soft-tissue mass originating from the calcaneus, permeative destruction of the entire bone with aggressive spiculated periosteal reaction and cortical destruction**.

**Figure 4:**
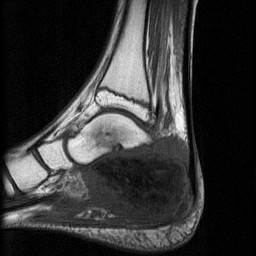
**MRI of the patient's foot shows a hypointense tumor mass on T1-weighted spin-echo sequences compared to surrounding musculature**. The skip lesion of the talus displays a hyposignal on T1-weighted sequences.

**Figure 5:**
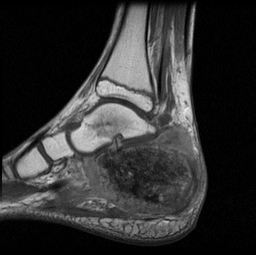
**MRI of the patient's foot shows a hyperintense tumor mass on T2-weighted spin-echo sequence images compared to surrounding musculature**. The skip lesion of the talus displays a hyposignal on T2-weighted sequences.

**Figure 6:**
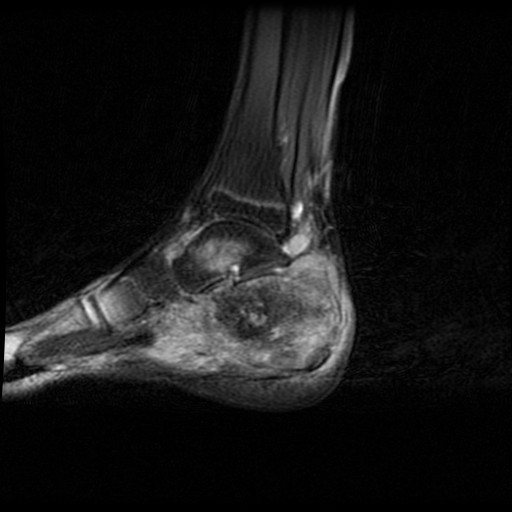
**T1-weighted fat saturation sequence after intravenous gadolinium chelate administration reveals strong contrast enhancement of the tumor**. We note the skip lesion in the talus as a low signal mass with peripheral enhancement.

Anteroposterior (Figure 1) and lateral radiographs (Figure 2) showed a condensed lesion in the calcaneus of his right foot with aggressive periosteal reaction and soft-tissue swelling. Computed tomography (CT) revealed a soft-tissue mass of the foot originating from his calcaneus and a sclerotic lesion of the entire bone with aggressive spiculated periosteal reaction and cortical destruction (Figure 3). A large soft-tissue mass around the involved bone was indicative of Ewing's sarcoma. Magnetic resonance imaging (MRI) was then performed and showed a hypointense tumor mass on T1-weighted sequences (Figure 4) and hyperintense properties on T2-weighted spin-echo sequences compared to surrounding musculature (Figure 5), a signal pattern characteristic of most tumors. The skip lesions of the talus displayed hyposignal properties on T1- and T2-weighted sequences. After intravenous gadolinium chelate administration, strong contrast enhancement of the tumor was observed (Figure 6). Skip metastases of the talus were evidenced as low-signal masses with peripheral enhancement (Figure 6).

A biopsy was performed and histopathology showed a malignant small round-cell tumor, identified as Ewing's sarcoma at immunohistochemistry study. Chest radiography and liver ultrasonography excluded the presence of any distant metastases. Our patient started neoadjuvant chemotherapy and underwent a below-knee amputation. Postoperative histology confirmed the diagnosis. Our patient remained disease-free for six months after diagnosis. Based on these findings, a diagnosis of Ewing's sarcoma of the calcaneus was made.

## Discussion

Ewing's sarcoma is a rare malignant neoplasm, predominantly affecting young patients of the ages five to 20 years. It involves the diaphyses of long bones and occurs less commonly in flat bones [1]. Clinical and laboratory features include local pain, soft-tissue swelling and erythema, occasionally accompanied with fever, anemia, leukocytosis, and accelerated erythrocyte sedimentation rate [2]. It rarely affects the feet.

Cook listed 29 cases of Ewing's sarcoma of the calcaneus in the literature since 1921 [3]. These rare cases are usually misdiagnosed, leading to treatment delay, which is detrimental to the outcome.

According to a retrospective study concerning 235 patients with non-metastatic Ewing's sarcoma of the bone, 15 patients were identified with a skip lesion at diagnosis. However, the skip lesions were located in adjacent juxta-articular bone in only two cases [4].

The radiographic features of Ewing's sarcoma in our case were those of classic Ewing's sarcoma: a permeative, lytic and condensed lesion with cortical destruction, aggressive periosteal reaction, large extraosseous soft-tissue component and often sclerotic reaction [5,6]. In spite of clinical and radiological findings, Ewing's sarcoma can be misinterpreted as osteomyelitis, cartilaginous tumor, giant cell lesion, lymphoma or osteosarcoma, and the distinction often requires extensive evaluation using varied imaging modalities [7].

CT can reveal a soft-tissue mass of the foot, such as permeative lytic lesions of the bone with aggressive periosteal reaction and cortical destruction, but the distinction between osseous remnants, reactive changes and tumor matrix can sometimes be challenging [8]. Bone scintigraphy of the whole skeleton demonstrates a focus of increased uptake of technetium-99 m-methylene diphosphonate [8,9].

T2-weighted MRI cannot adequately distinguish tumor from necrosis, and lesion boundaries are frequently overestimated because of the presence of edema and hemorrhage [9]. The enhancement pattern after administration of contrast medium on MRI allows differentiation between a tumor and peritumoral reactive edema. Furthermore, MRI can often distinguish the large solid sarcomatous soft-tissue mass around the involved bone from edema or an osteomyelitic abscess. MRI findings can narrow the differential diagnosis, but a specific diagnosis can rarely be established. Therefore, a biopsy of the tumor with histopathological analysis is needed to confirm the diagnosis. Staging, prior to biopsy, is essential to document the local and distant spread of the tumor. In Ewing's sarcoma, the metastatic pattern may be pulmonary involvement, bone or bone marrow spreading, skip metastases, or combined metastatic disease [7,9].

The imaging features of local spread of Ewing's sarcoma, involving small bones to adjacent bones, have not been described in the recent literature. It wasn't possible to determine the exact local extent of the tumor by means of conventional radiography and CT [5,9].

Due to its superior contrast resolution and multiplanar capabilities, MRI is more sensitive than other imaging techniques, especially for the investigation of tumor spread to bony structures and bone marrow. MRI should always be performed in the analysis of Ewing's sarcoma since it allows accurate evaluation of the tumor extent, which is decisive for treatment [10].

Skip lesions in patients with otherwise non-metastatic skeletal Ewing's sarcoma may be of the same importance as the molecular detection of marrow metastases, and possibly confer a worse prognosis. Newer imaging modalities like positron emission tomography-computed tomography and careful staging work-up may indicate that skip metastases in Ewing's sarcoma are more common than previously suspected [8,10].

## Conclusions

This case report confirms that the routine radiological management of Ewing's sarcoma should include radiography and MRI of the affected region, together with whole skeleton bone scintigraphy and CT of the chest. MRI is essential in the determination of the true extent of the tumor. It is important to bear in mind that early recognition of an unusual appearance and location of Ewing's sarcoma is necessary for its adequate treatment.

## Abbreviations

CT: computed tomography; MRI: magnetic resonance imaging

## Consent

Written informed consent was obtained from the father of our patient for publication of this case report and any accompanying images. A copy of the written consent is available for review by the Editor-in-Chief of this journal.

## Competing interests

The authors declare that they have no competing interests.

## Authors' contributions

HJ, ZB and HE made, analyzed and interpreted our patient's imaging examinations. MM and TF are the traumatologists whom operated on our patient and made major contributions to the manuscript. The manuscript was prepared by HJ under the supervision of OE and AO. All authors read and approved the final manuscript.
